# 

**Published:** 1840-01

**Authors:** 


### WESTERN JOURNAL

OF

### MEDICINE AND SURGERY:

EDITED BY

DANIEL DRAKE, M. D.

AND

LUNSFORD P. YANDELL, M. D.

PROFESSORS IN THE LOUISVILLE MEDICAL INSTITUTE.

NO. I.—JANUARY, 1840.

LOUISVILLE, KY.

PUBLISHED MONTHLY, BY PRENTICE & WEISSINGER,

PROPRIETORS AND PRINTERS.

1840.

